# Influence of hydroxyapatite nanoparticles on the formation of calcium fluoride surface layer on enamel and dentine in vitro

**DOI:** 10.1038/s41598-022-21545-1

**Published:** 2022-10-20

**Authors:** Tina Rodemer, Norbert Pütz, Matthias Hannig

**Affiliations:** Clinic of Operative Dentistry, Periodontology and Preventive Dentistry, Saarland University Hospital, 66421 Homburg, Saarland Germany

**Keywords:** Health care, Medical research

## Abstract

Topical application of different fluoride preparations is considered to be the gold standard of oral prophylaxis measures in preventive dentistry. Hydroxyapatite nanoparticles (nano-HAP) as well, have received considerable attention for dental use in the past few decades. The purpose of this in-vitro study was to analyze the interaction between nano-HAP and different fluoride preparations. In order to investigate the possibility to establish, in the presence of nano-HAP, reproducible calcium fluoride surface layers, specimens were visually examined with regard to the surface coverage’s structure, morphology, homogeneity and stability. Test series based on enamel and dentine specimens, that were obtained from extracted bovine teeth, were conducted. Thereby, sodium fluoride, olaflur, elmex Fluid (10.000 ppm) and an aqueous nano-HAP solution (5%) served as test products and sterile water as reference. First, single application of nano- HAP and fluoride was tested. After 5 min of incubation in the test solution, the surface coverage was examined by scanning electron microscopy (SEM). Furthermore, samples were determined by energy dispersive X-ray spectroscopy (EDX) to identify the present elements of the surface layer, particularly fluoride. To test the calcium fluoride layer’s persistence and stability, samples were exposed to the spray of a dental multifunctional syringe for 20 s using maximum pressure and maximum water supply. In the second application protocol, fluoride and nano-HAP were applied simultaneously and in the third application protocol they were used sequentially. SEM visualisation showed that the simultaneous or sequential addition of nano-HAP led to a distinct change in the surface layer’s structure. Agglomerates of various sizes were formed, with obviously different morphology from the calcium fluoride globules, not covering the surface homogeneously and sprayed off with the multifunctional syringe easily. Application of pure fluoride compounds resulted in a more homogeneous calcium fluoride surface layer with higher persistence in comparison to the combination of fluoride and nano-HAP. Interaction between fluoride and nano-HAP clearly could be proved. On enamel as well as dentine surfaces, the combined application of nano-HAP and fluoride has a negative effect on the stability and persistence of the calcium fluoride surface precipitate.

## Introduction

A pivotal component of health maintenance of either a natural healthy or caries-free rehabilitated dentition are fluoridation measures for caries prophylaxis^1^, with consistent application of toothpaste containing fluoride being the focus^2,3^. In addition to toothpastes, fluoride-containing varnishes, gels and rinsing solutions that are applied topically are used to prevent tooth decay^4–6^.

The key mechanism of action of fluorides is their accumulation on dental hard tissue in the form of calcium fluoride or calcium fluoride-like precipitates configuring a calcium fluoride globule layer^7,8^.

The use of biomimetic substances for oral care has received considerable attention in preventive dentistry. Calcium phosphates represent a biomimetic alternative because of their high similarity to the mineral phase of the teeth^9^. Of all calcium phosphates, hydroxyapatite is most similar to the apatite crystals of enamel. In addition, hydroxyapatite has the lowest solubility of all calcium phosphates and can be synthesized in various crystal structures and particle sizes, for example from micrometers to nanometers^10^.

The interactions between nano-HAP particles and the enamel surface, which lead to remineralization, are not known in detail, yet^11^. A possible mechanism that is discussed is that the nano-HAP particles induce remineralization by acting as a nucleus that attracts calcium and phosphate from the saliva^12^. Numerous studies have examined the qualitative interaction of nano-HAP with enamel and the pellicle^11,13–17^. Fabritius-Vilpoux et al*.* also considered quantitative parameters of the affinity of nano-HAP for enamel such as concentration and particle size^13^. The authors used standardized bovine enamel test specimens under in vitro conditions to investigate the interactions between nano-HAP and enamel without the influence of individual patient-related parameters. The authors concluded that with increasing concentration of the nano-HAP particles, more enamel surface is covered and that smaller particles adhere better to the enamel surface than larger ones.

Ebadifar et al*.*^18^ investigated in vitro the effect of a toothpaste with fluoride and nano-HAP compared to a pure fluoride toothpaste regarding the microhardness of artificial carious lesions. Thus, the authors concluded that the mixture of nano-HAP and fluoride has a higher remineralizing effect than the pure fluoride toothpaste and therefore suspect a synergistic effect of the two substances.

Soares et al*.*^19^, however, came to the opposite conclusion. They investigated the anti-erosive effect of an APF gel (acidulated phosphate fluoride) with nano-HAP on the enamel surface. Treatment with APF gel resulted in mineral gain after the erosion process. Treatment with APF gel containing nano-HAP, on the other hand, led to a significant loss of phosphate. As a result, nano-HAP has a negative impact on enamel protection when it is directly combined with fluoride. One possible explanation of the authors is that the addition of nano-HAP to an acidic fluoride gel results in partial dissolution of the nano-HAP. As a result, the released calcium binds the fluoride in the gel. In this way, both substances are ultimately inactivated.

Nevertheless, little is known about the interaction of the topical application of nano-HAP and fluorides. The question arises whether the two substances interact when applied simultaneously or sequentially by having a synergistic effect or whether nano-HAP has a inhibiting effect on the establishment of a calcium fluoride surface layer, possibly even prevents it.

## Materials and methods

### Preparation of specimens

This in-vitro experiment was conducted with 144 square enamel and dentine slabs in total gained from labial surfaces of bovine incisors of 2-year-old freshly slaughtered cattle from the slaughterhouse Zweibrücken, Germany. First, the roots of the extracted teeth were cut off with a diamond disc under water cooling, the crowns were cut in the frontal plane and the specimen blanks (6 mm × 4 mm × 3 mm) were cut out of the labial surfaces.

The surfaces were wet-ground and polished in a standardized grinding procedure with up to 2500 grid abrasive paper (Silicon Carbide Grinding Paper, Buehler, Illinois, USA) and the resulting smear layer was removed according to the following standardized cleaning procedure. First, enamel samples were washed with 3% NaOCl for 2 min, dentine samples for 30 s followed by ultrasonication with distilled water for 5 min (enamel) and 2 min (dentine). Next, samples were disinfected in 70% ethanol for 15 min, washed in distilled water and finally stored at 4ºC in fresh distilled water for 24 h.

### Experimental solutions

Fluoride treatments were performed using sodium fluoride as an inorganic fluoride preparation (reinst Ph. Eur., Ferdinand Kreutzer Sabamühle GmbH, Nürnberg, Germany) and olaflur (Permafluor, Permcos GmbH, Stein, Switzerland) as an organic fluoride preparation. Aqueous solutions containing 10.000 ppm F^−^ were prepared with distilled water (Ampuwa^®^, Fresenius Kabi Deutschland GmbH, Bad Homburg, Germany). Elmex Fluid (CP GABA GmbH, Hamburg, Germany), a commercially available amine fluoride solution (9250 ppm Olaflur and 750 ppm Dectaflur) was used as well. The nano-HAP solution was prepared by mixing 5 g nano-HAP (Kalident 100, Kalichem, Brescia, Italien) with 100 ml distilled water.

Additionally, aqueous test solutions containing both, fluoride and nano-HAP, were prepared by mixing either sodium fluoride, Olaflur or elmex Fluid (10.000 ppm F^−^) with nano-HAP (5%) and 100 ml distilled water.

### Application protocol

**Figure 1:**
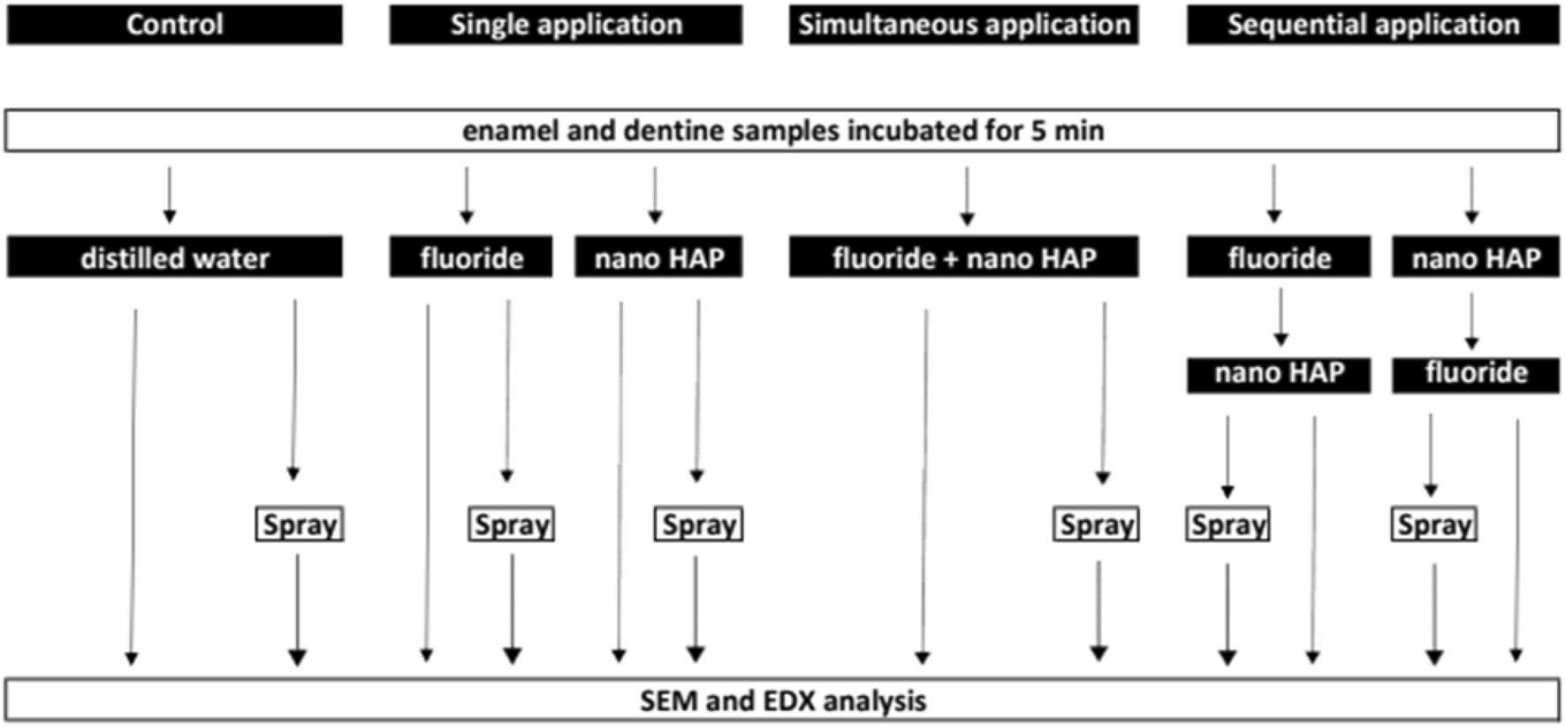
Flow chart of the application protocols fluoride = sodium fluoride, olaflur or elmex Fluid. *Nano-HAP* hydroxyapatite nanoparticles.

Three different application protocols were conducted to evaluate the effect of nano-HAP on the formation of calcium fluoride surface layers on enamel and dentine in vitro (Fig. 1).

In the first application protocol, the single application of nano-HAP (pH  5.38), sodium fluoride (pH  5.38), olaflur (pH  5.25) and elmex Fluid (pH  4.50) was tested, respectively. Sterile water (pH  5.73) served as control. After incubation for 5 min in 1.000 μl of the test solution, the samples surface coverage was examined by scanning electron microscopy (SEM) to visualize the structure, morphology and homogeneity and by energy dispersive X-ray spectroscopy (EDX) to detect fluoride. In order to test persistence and stability of the surface layer, samples were exposed to the spray of a dental multifunctional syringe for 20 s using maximum pressure and maximum water supply. Subsequently, SEM and EDX analysis were performed again.

In the second application protocol, fluoride and nano-HAP were applied simultaneously as combination of sodium fluoride + nano-HAP (pH  5.38), olaflur + nano-HAP (pH  6.51) and elmex Fluid + nano-HAP (pH  6.00), respectively. SEM and EDX analysis were performed. In the second part, samples were sprayed off before analyzed by SEM and EDX.

In the third application protocol, fluoride and nano-HAP were used sequentially in two ways. First, fluoride application was followed by nano-HAP and then the other way round. Samples were also sprayed off and analyzed by SEM and EDX. Each time, the experimental setup was the same.

In each individual experimental subgroup three enamel and three dentine specimens were treated according to the various application protocols (Fig. 1).

### Scanning electron microscopy and EDX analysis

After overnight airdrying at room temperature in the air chamber, samples were fixed to SEM sample holders (aluminium plate) and sputter-coated with carbon. In order to characterize the surface coverages, samples were analyzed by SEM at 5 kV at up to 20,000-fold magnification and an element analysis was determined by EDX at 10 kV at 500-fold magnification in a XL 30 ESEM FEG (FEI, Eindhoven, The Netherlands). The weight percentage of fluorine was determined by EDX at five different surface regions (measuring 200 × 300 µm each) on each individual enamel and dentine slab. For each experimental subgroup means and standard deviations for fluorine weight percentage were calculated. Statistical analysis was performed by one-way ANOVA using GraphPad Prism 9.4.1. The adjusted p-value for significant differences between experimental groups was set at p < 0.0001.

## Results

### Control

**Figure 2:**
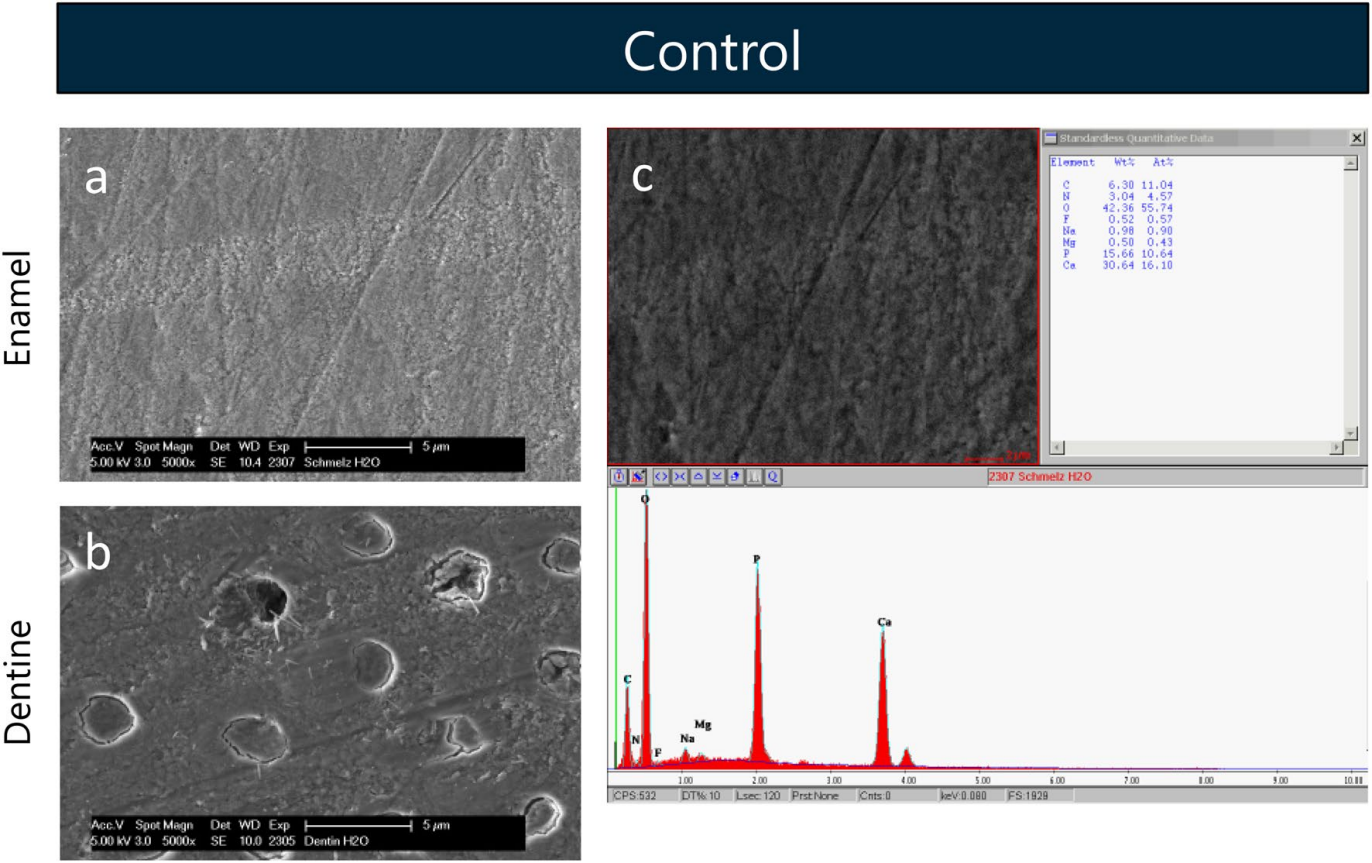
SEM figures at 5000-fold magnification of enamel (**a**) and dentine (**b**) samples and exemplaric EDX analysis of a enamel sample (**c**) after 5 min incubation in distilled water. The images reveal a polished enamel and dentine surface without any precipitates.

Sterile water was used as negative control. The SEM images showed a typical surface of polished, untreated enamel (Fig. 2a) or dentine (Fig. 2b) without any precipitates and served as reference for morphological changes. The fluoride weight percent (F[Wt%]) of the reference samples determined by EDX analysis (Fig. 2c) was < 1% (Table 1).

**Table 1:** Results of EDX analysis of weight percent fluorine (F[Wt%]).

	Control	Single application	Simultaneous application	Sequential application
**Enamel specimens**				
	Distilled water < 1.0	Nano HAP < 1.0		
		NaF 6.40 ± 1.19	NaF + nano HAP 9.23 ± 5.26	NaF / nano HAP 1.83 ± 0.45 Nano HAP / NaF 5.33 ± 1.44
		Olaflur 24.22 ± 0.62	Olaflur + nano HAP 10.95 ± 3.51	Olaflur / nano HAP 16.56 ± 4.7 Nano HAP / Olaflur 19.09 ± 5.4
		Elmex 29.2 ± 0.81	Elmex + nano HAP 11.67 ± 2.03	Elmex / nano HAP 9.19 ± 5.54 Nano HAP / Elmex 23.26 ± 7.74
Sprayed	Distilled water < 1.0	Nano HAP < 1.0		
Sprayed		NaF 5.15 ± 0.5	NaF + nano HAP 1.09 ± 0.09	NaF / nano HAP 4.83 ± 0.79 Nano HAP / NaF 2.06 ± 0.99
Sprayed		Olaflur 23.14 ± 0.94	Olaflur + nano HAP 0.96 ± 0.21	Olaflur / nano HAP 17.56 ± 1.21 Nano HAP / Olaflur 16.3 ± 4.97
Sprayed		Elmex 29.49 ± 0.94	Elmex + nano HAP 1.06 ± 0.19	Elmex / nano HAP 31.1 ± 0.48 Nano HAP / Elmex 19.55 ± 12.05
**Dentine specimens**				
	Distilled water < 1.0	Nano HAP < 1.0		
		NaF 10.34 ± 0.90	NaF + nano HAP 9.53 ± 3.58	NaF / nano HAP 1.73 ± 0.72 Nano HAP / NaF 6.16 ± 0.85
		Olaflur 18.68 ± 0.47	Olaflur + nano HAP 11.12 ± 3.15	Olaflur / nano HAP 7.76 ± 4.86 Nano HAP / Olaflur 18.22 ± 1.34
		Elmex 20.68 ± 0.25	Elmex + nano HAP 8.55 ± 4.11	Elmex / nano HAP 11.27 ± 5.42 Nano HAP / Elmex 12.57 ± 3.89
Sprayed	Distilled water < 1.0	Nano HAP < 1.0		
Sprayed		NaF 5.44 ± 1.14	NaF + nano HAP 1.46 ± 0.13	NaF / nano HAP 5.82 ± 2.82 Nano HAP / NaF 6.05 ± 0.81
Sprayed		Olaflur 16.77 ± 0.83	Olaflur + nano HAP 1.45 ± 0.23	Olaflur / nano HAP 14.51 ± 2.53 Nano HAP / Olaflur 14.85 ± 1.38
Sprayed		Elmex 20.97 ± 0.5	Elmex + nano HAP 1.62 ± 0.14	Elmex / nano HAP 23.21 ± 0.42 Nano HAP / Elmex 18.78 ± 2.35

Measurements were performed after 5 min incubation in the test solution (distilled water, fluoride, nano-HAP, fluoride + nano-HAP) and after 20 s spraying with the multifunctional syringe (sprayed) according to the different application protocols. Fluoride = sodium fluoride, olaflur and elmex Fluid.

*Nano-HAP *hydroxyapatite nanoparticles.

### Single application

**Figure 3:**
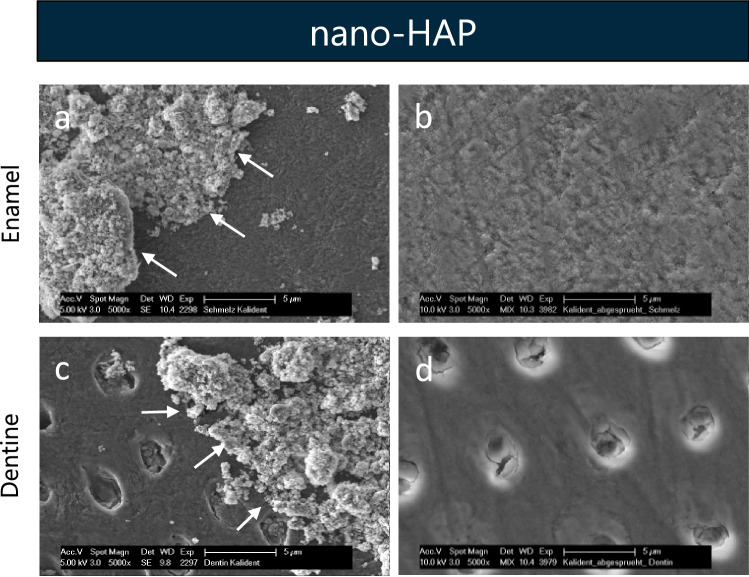
SEM figures at 5000-fold magnification of enamel (**a,b**) and dentine (**c,d**) samples after 5 min incubation in aqueous nano-HAP solution (**a,c**) and after 5 min incubation in aqueous nano-HAP solution and subsequent 20 s spraying with the multifunctional syringe (**b,d**). The images reveal nano-HAP particles of different size and shape. White arrows point to nano-HAP particles accumulated onto the surface. After spraying, a polished enamel and dentine surface without any precipitates is visible. *Nano-HAP* hydroxyapatite nanoparticles.

After the application of the nano-HAP suspension, crystalline deposits could be detected on the enamel (Fig. 3a) and dentine (Fig. 3c) surfaces. The particles and clusters of different shape and size were inhomogeneously distributed over the entire surface.

After exposing the specimens to the spray of a dental multifunctional syringe, no surface coverage was detected neither on enamel (Fig. 3b) nor on dentine (Fig. 3d). The SEM images of the samples revealed the typical morphology of polished tooth surfaces. No more precipitates could be seen. The EDX analysis determined a weight percent of fluoride (F[Wt%]) < 1% (Table 1).

**Figure 4:**
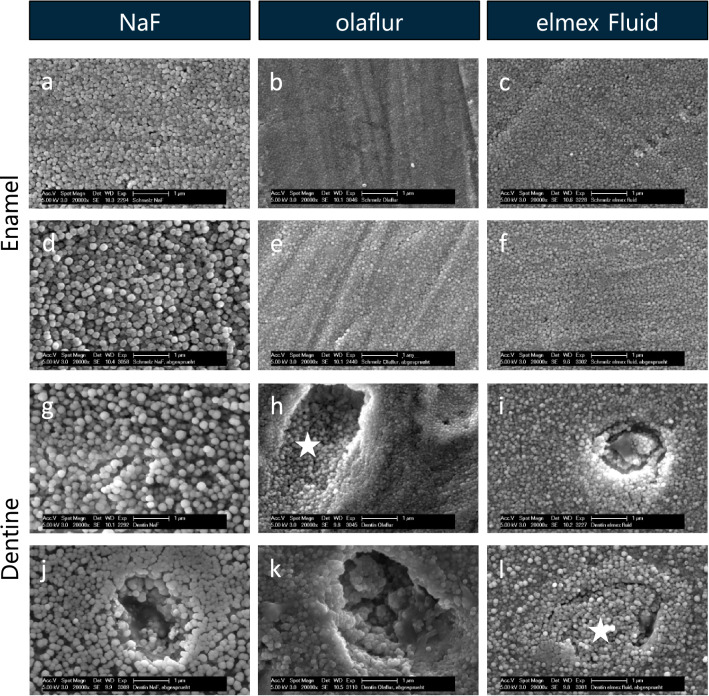
SEM figures at 20,000-fold magnification of enamel (**a–f**) and dentine (**g–l**) samples after 5 min incubation in sodium fluoride (**a,g**), olaflur (**b,h**), elmex Fluid (**c,i**) and subsequent 20 s spraying with the multifunctional syringe (**d–f,j–l**). The images reveal a complete masking of the surface with globular precipitates, even after spraying. White asterisks mark dentine tubules coated with globules. *NaF* sodium fluoride.

After treatment according to the first application protocol of fluorides, SEM images of the enamel (Fig. 4a) and dentine (Fig. 4g) surfaces treated with sodium fluoride showed a very homogeneous coverage with spherical precipitates. After the application of Olaflur (Fig. 4b,h) and elmex Fluid (Fig. 4c,i), more tightly and smaller globules could be seen. On dentine, the fluoride cover layer partially coated the walls of transversely cut dentine tubules at the lumen (Fig. 4h), other tubules seemed to be superficially sealed by the fluoride layer (Fig. 4i).

In the second part of the first application protocol, test specimens were exposed for 20 s to the spray of a multifunctional syringe under maximum pressure and maximum water supply. The SEM images barely differed from those of the previous test series without spraying. A homogeneously fluoridated surface impressed on both enamel and dentine, even after spraying (Fig. 4d–f,j–l).

The fluoride mean weight percentages (F[Wt%]) determined by EDX of the samples after single application of different fluorides ranged from 6.4 to 29.2% on enamel and from 10.3 to 20.7% on dentine specimens, and were not significantly changed due to the water spraying (Table 1). Significantly (p < 0.0001) highest percentages of fluoride were obtained after application of elmex Fluid, whereas significantly (p < 0.0001) lowest percentages of fluoride were detected after treatment with sodium fluoride.

### Simultaneous application

**Figure 5:**
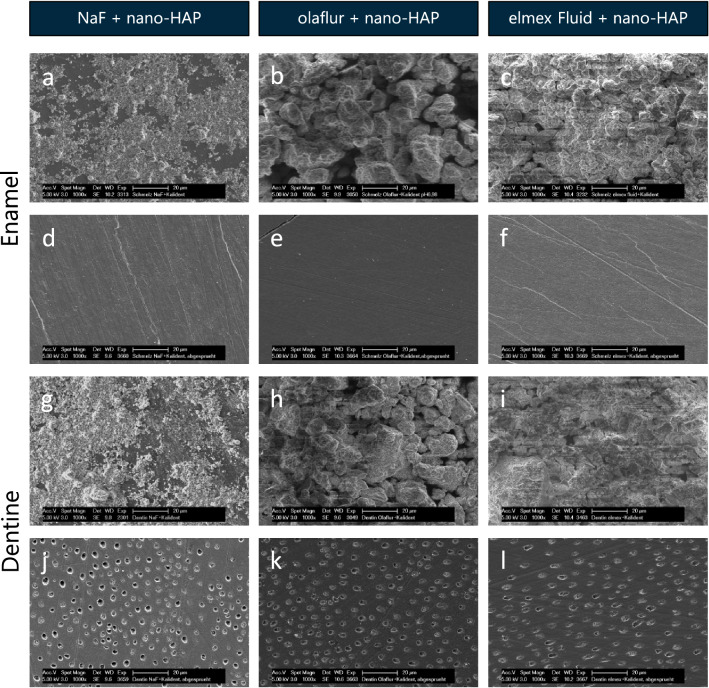
SEM figures at 1000-fold magnification of enamel (**a–f**) and dentine (**g,l**) samples after 5 min incubation in aqueous nano-HAP solution combined with sodium fluoride (**a,g**), olaflur (**b,h**), elmex Fluid (**c,i**) and subsequent 20 s spraying with the multifunctional syringe (**d–f,j–l**). The images reveal nano-HAP agglomerates masking the surface, which neither resemble the nano-HAP particles nor the calcium fluoride globules. After spraying, a polished enamel and dentine surface without any precipitates is visible. *Nano-HAP* hydroxyapatite nanoparticles, *NaF* sodium fluoride.

After the second application protocol, the simultaneous application of both sodium fluoride and nano-HAP on enamel (Fig. 5a) or dentine (Fig. 5g), a very homogeneous, dense coverage of the sample surface resulted with individual larger agglomerates that were slightly lighter than the background. After the application of both, olaflur (Fig. 5b,h) or elmex Fluid (Fig. 5c,i) combined with nano-HAP, large agglomerates were observed, which in terms of their shape and structure resemble neither the nano-HAP particles nor the fluoride globules. After the mixtures had been sprayed off, no fluoride-containing top layer could be seen. There was no surface coverage at all on either enamel or dentine (Fig. 5d–f,j–l).

After simultaneous application of sodium fluoride and nano-HAP on enamel or dentine the mean fluoride weight percent (F[Wt%]) was 9.2% and 9.5%, respectively, and did not differ significantly from the fluoride mean weight percentage after single application of sodium fluoride without additional nano-HAP (Table 1). In contrast, treatment with olaflur or elmex Fluid combined with nano-HAP resulted in significantly (p < 0.0001) lower mean fluoride weight percentage compared to the single application of olaflur or elmex Fluid (Table 1). After water spraying of the samples treated simultaneously with fluoride and nano-HAP the mean weight percentage (F[Wt%]) of fluoride was significantly (p < 0.0001) reduced to around 1% on enamel, and around 1.5% on dentine specimens (Table 1). These fluoride values were significantly (p < 0.0001) lower compared to the enamel and dentine specimens solely treated with the various fluoride solutions and processed to water spraying (Table 1).

### Sequential application

**Figure 6:**
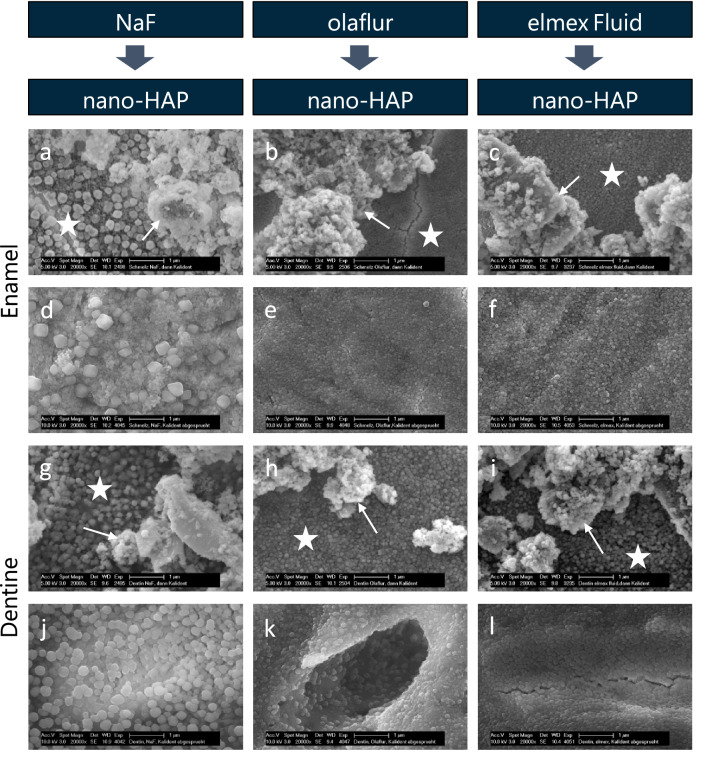
SEM figures at 20,000-fold magnification of enamel (**a–f**) and dentine (**g–l**) samples after 5 min primary incubation in sodium fluoride (**a,g**), olaflur (**b,h**), elmex Fluid (**c,i**) and 5 min secondary incubation in aqueous nano-HAP solution and subsequent 20 s spraying with the multifunctional syringe (**d–f,j–l**). The images reveal an inhomogenous coverage of the fluoridated surface with nano-HAP particles of different size and shape. White asterisks mark the surface masked with globules. White arrows point to accumulated nano-HAP particles with the tendency for clusters formations. After spraying, a surface homogenously covered with globular precipitates is visible. The nano-HAP particles are completely washed off. *Nano-HAP* hydroxyapatite nanoparticles, *NaF* sodium fluoride.

After the sequential application of first fluoride and then nano-HAP, beside smaller and larger nano-HAP agglomerates, fluoridated surfaces without deposited particles were visible (Fig. 6a–c,g–i). The tendency for formation of clusters could be observed in the samples treated sequentially. The primary fluoridation did not affect the deposition of nano-HAP.

The nano-HAP particles are completely washed off by exposure to the spray of the dental multifunctional syringe. A surface, densely covered with spherical precipitates was visualized on enamel as well as on dentine (Fig. 6d–f,j–l).

Sequential application of fluoride first followed by nano-HAP resulted in significantly (p < 0.0001) reduced mean values of fluorine weight percent (F[Wt%]) as compared to the specimens treated with fluoride only. Interestingly, after water spraying, the fluoride values of the enamel and dentine specimens, that were treated sequentially with fluoride followed by nano-HAP, increased significantly (p < 0.0001) and were in the same range as the mean weight percentage (F[Wt%]) of fluoride after single application of the three fluoride solutions and subsequent spray treatment (with one exception: in case of the olaflur treated enamel the fluoride mean value was significantly lower in the sequentially treated compared to the solely treated slab), (Table 1).

**Figure 7:**
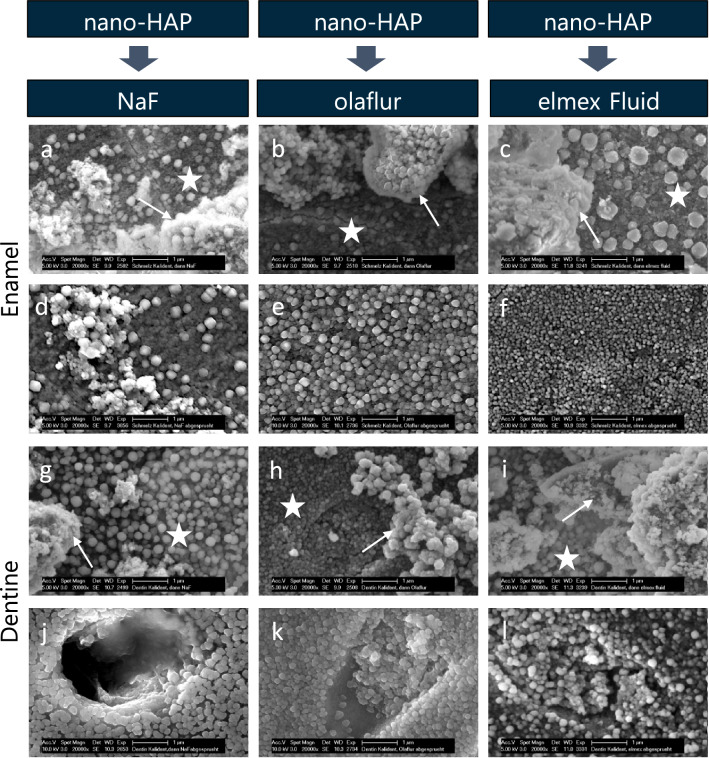
SEM figures at 20,000-fold magnification of enamel (**a–f**) and dentine (**g–l**) samples after 5 min primary incubation in aqueous nano-HAP solution and 5 min secondary incubation in sodium fluoride (**a,g**), olaflur (**b,h**), elmex Fluid (**c,i**) and subsequent 20 s spraying with the multifunctional syringe (**d–f,j–l**). The images reveal an inhomogenous coverage of the fluoridated surface with nano-HAP particles of different size and shape. White asterisks mark the surface masked with globules. White arrows point to accumulated nano-HAP particles masked with calcium fluoride globules. After spraying, a surface homogenously covered with globular precipitates is visible. The nano-HAP particles are completely washed off. *Nano-HAP* hydroxyapatite nanoparticles, *NaF* sodium fluoride.

Following the third application protocol, after primary incubation in nano-HAP and secondary fluoridation, individual globules could be found masking the nano-HAP particles and adjacent, smaller spherical precipitates covered the tooth surface (Fig. 7a–c,g–i).

After water rinsing the sodium fluoride nano-HAP treated enamel and dentine samples, the surface was covered with fewer, loosely distributed globules and only remnants of particulate structures (Fig. 7d,j). Nano-HAP clusters could no longer be visualized on the SEM images of the olaflur and elmex Fluid samples, but a dense, homogeneous surface coverage with spherical precipitates (Fig. 7e,f,k,l). Nano-HAP does not affect the fluoridation here.

Primary application of nano-HAP followed by fluoridation resulted in higher fluoride values as compared to specimens treated with fluoride first and secondary application of nano-HAP. However, these differences were only significant (p < 0.0001) in case of enamel samples fluoridated with elmex Fluid after nano-HAP pretreatment, and after secondary application of NaF or olaflur in case of primary nano-HAP incubated dentine slabs. After water spraying, the fluoride values of the nano-HAP and subsequently fluoride treated specimens were in the same range as the *vice versa* treated samples. Only in case of the specimens treated with elmex Fluid, water rinsing caused a significant (p < 0.0001) reduction of the fluoride weight percent (F[Wt%]) regarding the enamel as well as dentine specimens treated with nano-Hap first compared to the secondary application of nano-HAP.

### EDX analysis

EDX analysis was performed to detect the present elements in the surface layer. The aim of the EDX analysis was to prove that after single, simultaneous and sequential application of different fluoride containing solutions, fluorine is effectively verifiable in the precipitates. Further, possible alterations by addition of nano-HAP were revealed. The results are summarized in Table 1.

## Discussion

The present in-vitro investigation clearly demonstrated the interactions between nano-HAP and fluoride preparations. The presence of nano-HAP modified the calcium fluoride surface layer’s structure, morphology, homogeneity and stability on enamel and dentine.

In this in-vitro study, bovine teeth were used for the preparation of specimens. The use of human teeth is limited and has some disadvantages. It is often difficult to get them in sufficient quantity and quality because most of the teeth are extracted due to extensive carious lesions or other defects. The structure and composition of human teeth is age-dependent and individually different, leading to different study results.

Among dental hard tissue of non-human origin, bovine samples are the most preferred substitute^20^ because of their good availability, uniform composition and comparability with human teeth, especially with regard to calcium content^13,21^. The calcium content of bovine teeth is almost identical to that of human teeth and shows a gradual decrease from the enamel to the enamel-dentine junction^22^. Furthermore, the amino acid composition of bovine enamel matrix is similar to that of human enamel matrix^23^. Another difference, particularly important for the present study, is the fluoride content. Bovine teeth have a lower natural fluoride concentration^20^, which has the advantage, that the fluoride determined by EDX is predominant due to the topical applied fluoride. In our experiments, the natural fluoride concentration of bovine specimens was 0.60% at the maximum.

Using SEM in combination with EDX to characterize fluoride precipitates has already been published^24^. Scholz et al*.*^25^ evaluated in vitro superficial calcium fluoride-precipitation on human enamel after treatment with different fluoride-containing gels using EDX. The result of this study was that EDX is a reliable and relatively fast method to semiquantitatively measure calcium fluoride-precipitates on tooth surfaces. The advantage of semiquantitative analysis using EDX is that, in addition to information on fluoride concentration, SEM analysis provides information about the morphology and distribution of the precipitates as well. Using EDX, detected elements can be directly assigned to specific locations on the samples’ surface.

In the present study, the application of sodium fluoride, olaflur and elmex Fluid with an exposure time of 5 min led to the formation of a homogenous calcium fluoride globule layer covering the entire surface on both enamel and dentine surfaces. Remarkably, the fluoride content on enamel after using the amine fluoride preparations olaflur and elmex Fluid is significantly higher than after application of sodium fluoride with same initial concentration (10,000 ppm each). Similar results were obtained in other studies under both in-vitro and in-situ conditions^8,26,27^. Dentine samples treated with sodium fluoride showed the lowest fluoride content as well. However, this is almost twice as high as the corresponding content on enamel. This result only partially coincides with results from other studies^8^.

If the layer of calcium fluoride globules is established by using amine fluorides, smaller globules will be formed than by using sodium fluoride. These globules adhere very well to the tooth surface^28^ and build a stable fluoride reservoir there^29^. As observed in the present study, globules had a smaller diameter, around 0.2 µm, after treatment with olaflur and elmex Fluid than after treatment with sodium fluoride (0.1 µm) as well. The formation of globules with a fine grain size is facilitated by the surface activity of amine fluorides and their weakly acidic pH value^30^. An important factor for the establishment of the calcium fluoride globule layer is the availability of calcium ions originated from saliva, plaque or enamel^24^. The availability of ions dissolved from enamel depends on the pH value. Thus, only a low pH value leads to a high enamel solubility^31^. Hence, the formation of the calcium fluoride layer also depends on whether the environment is acidic, neutral or basic^32^.

Further influencing factors, not considered in the present study, are the fluoride concentration and the influence of saliva^33–35^. All used fluoride preparations had a constant fluoride concentration of 10,000 ppm and neither natural nor artificial saliva was used.

For evaluation of salivary pellicle’s influence, Scholz et al*.*^25^ examined the fluoride precipitation on human enamel with and without pellicle. The pellicle reduced the fluoride content after treatment with acidic sodium fluoride gels significantly. Thus, fluoride values measured in vitro in absence of a pellicle might be misleadingly high. The authors explained this either by a barrier function of the pellicle or by an increase of pH value due to the pellicle on the enamel interface.

Various studies showed, that also nano-HAP particles adhere to the enamel surface^11,13–16,36–38^. However, in several studies under in vitro conditions, the enamel surface was covered with an artificial organic pellicle or nano-HAP particles were mixed with other organic substances or artificial saliva. Therefore, it is difficult to evaluate whether the nano-HAP particles adhere to enamel due to intrinsic mineral properties or because proteins or other organic molecules serve as connecting elements^13^. For this reason, Fabritius-Vilpoux et al*.*^13^ used cleaned enamel specimens and an aqueous dispersions of chemically pure synthetic HAP particles with neutral pH value and investigated in vitro, if the adhesion is based on sole mineral–mineral interactions. The authors found a dose–response relationship with increasing HAP concentration of 1–10% and that only particles with a diameter of approximately 1·3 µm did adhere to the enamel surface, whereas larger particles did not adhere^13,14^. In the present study, also both cleaned enamel and dentine specimens and aqueous HAP dispersion were used. Our own results show that treatment with nano-HAP dispersion led to an inhomogeneous coverage of the tooth surface, which is reflected in the results by Fabritius-Vilpoux et al*.* In the present experimental setup, the nano-HAP particles could be completely removed simply by spraying with the multifunctional syringe under full pressure and full water supply. Thus, electrostatic forces, van der Waals forces and hydrogen bonds between nano-HAP particles and enamel are not sufficient to provide a stable and permanent surface coverage. Therefore, it can be suggested that the pellicle has greater influence on the adhesion than the chemical bonding forces mentioned above^13^. Because of connective structures between the pellicle and nano-HAP particles observed in situ it would be interesting to repeat the present experimental setup under oral conditions in a follow-up study^39^.

Concerning the combined preparations containing both nano-HAP and fluoride several studies investigated the anti-erosive effects on enamel as well as on dentine in vitro. Preparations with both nano-HAP and fluoride were not superior to comparing preparations containing sodium or amine fluoride^40–43^. After application of a toothpaste containing 7% HAP and 1000 ppm sodium fluoride the increase in microhardness after acid exposure was higher than after using a toothpaste just with sodium fluoride. The authors conclude that the mixture of both HAP and fluoride has a higher remineralizing effect than pure fluoride toothpaste and suspect a synergistic effect of the two substances^18^. However, there is the opposite conclusion that both substances are ultimately inactivated. This is a result of an investigation of an APF (acidulated phosphate fluoride) gel with or without HAP. The authors describe a negative influence on enamel protection when HAP is combined directly with fluoride^19^.

The agglomerates detected in the present study after application of the nano-HAP-fluoride mixtures have not been described in the scientific literature, yet. They refer to a possible reaction between nano-HAP and fluoride. Since neither the exact chemical composition nor the biochemical processes were investigated in the present study, no statement can be made about whether the interaction already takes place during the production of the nano-HAP-fluoride mixtures or just when they come into contact with the tooth surface. The easy removal by spraying with the multifunctional syringe indicates a reaction between the two components and no interaction with enamel or dentine. So, the obvious assumption is that the solid bond of fluoride to the tooth surface is inactivated by adding nano-HAP. Thus, the results generally correspond to the results mentioned above, even if the experimental setup was not the same. Based on our own results, inactivation of nano-HAP and fluoride is more likely than the assumed synergistic effect.

**Figure 8:**
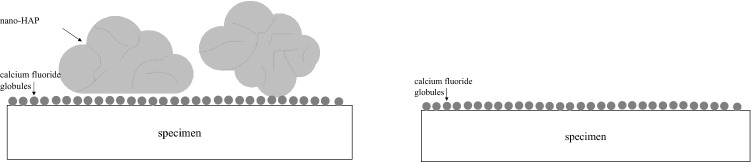
Schematic figure after primary fluoride application and subsequent nano-HAP application. Primary fluoridation does not compromise nano-HAP adsorption. Left: nano-HAP particles partially cover the calcium fluoride globules. Right: after spraying, nano-HAP particles are completely removed and a surface covered with calcium fluoride globules remains. *Nano-HAP* hydroxyapatite nanoparticles.

After primary fluoridation and subsequent incubation in the nano-HAP suspension, fluoride did not impair the deposition of nano-HAP on the tooth surface (Fig. 8). Morphologically, there is a homogenous fluoridated surface with superimposed nano-HAP particles. However, the fluoride content determined by EDX of both enamel and dentine specimens is lower than the value of the solely fluoridated specimens. A possible reason could be that the calcium fluoride globules are covered by nano-HAP particles and therefore, they are not detected by EDX analysis. After spraying the samples with the multifunctional syringe, nano-HAP particles are completely washed off, which indicates that there is no solid compound to the tooth surface.

**Figure 9:**
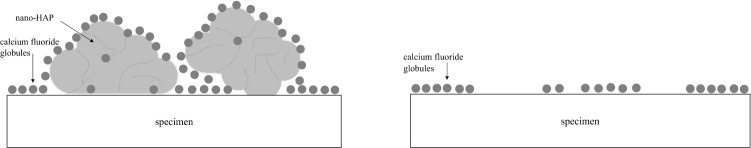
Schematic figure after primary nano-HAP application and subsequent fluoride application. Nano-HAP slightly compromises fluoridation. Left: nano-HAP particles are masked by calcium fluoride globules. Right: after spraying, nano-HAP particles are completely removed and a surface inhomogeneously covered with calcium fluoride globules remains. *Nano-HAP* hydroxyapatite nanoparticles.

Samples primarily treated with nano-HAP show nano-HAP particles masked by calcium fluoride globules (Fig. 9) and thus, they are included in the EDX analysis. Because of the great variety in shape and size of the nano-HAP clusters, the influence of the fluoride content differs a lot and so, higher and lower amounts are determined. After spraying secondarily fluoridated samples, nano-HAP clusters with superimposed calcium fluoride globules are entirely removed, because there is no resilient connection to the enamel or dentine surface.

Comparable studies investigating the sequential application of fluoride preparations and nano-HAP are not found in the literature.

## Conclusion

The reproducible establishment of homogeneous calcium fluoride surface layers with different fluoride compounds on enamel and dentine surfaces could be demonstrated. The presence of nano-HAP modified the surface coverage’s structure, morphology, homogeneity and stability on enamel and dentine.

Simultaneous application of fluoride and nano-HAP in a combined form proved interactions between nano-HAP and fluoride. The resulting surface coverage differs significantly from a calcium fluoride surface layer. After sequential application of fluoride and nano-HAP consecutively, nano-HAP did not modify the formation of the calcium fluoride surface layer.

The pure fluoride surface layers proved to be resistant. Nano-HAP, on the other hand, could be completely removed. Thus, the combined application of nano-HAP and fluoride has a negative effect on the stability and persistence of the calcium fluoride surface precipitate.

In conclusion, both the simultaneous and the sequential application of nano-HAP has a distinct influence on the fluoridation of enamel and dentine surfaces in vitro.

## Data Availability

All data generated or analysed during this study are included in this published article.
